# Mood instability and low back pain: a mendelian randomization study

**DOI:** 10.3389/fneur.2023.1252329

**Published:** 2023-09-15

**Authors:** Renyang Liu, Qian Liu, Shaoyong Xu, Rongcheng Mei

**Affiliations:** ^1^Department of Orthopaedics, Xiangyang Central Hospital, Affiliated Hospital of Hubei University of Arts and Science, Xiangyang, China; ^2^Center for Clinical Evidence-Based and Translational Medicine, Xiangyang Central Hospital, Affiliated Hospital of Hubei University of Arts and Science, Xiangyang, China; ^3^Department of Neurology, Xiangyang Central Hospital, Affiliated Hospital of Hubei University of Arts and Science, Xiangyang, China; ^4^Department of Endocrinology, Xiangyang Central Hospital, Affiliated Hospital of Hubei University of Arts and Science, Xiangyang, China

**Keywords:** mendelian randomization, low back pain, mood instability, genome-wide association, summary statistics

## Abstract

**Objective:**

Low back pain is a prevalent and debilitating condition worldwide, with significant implications for individuals’ quality of life and productivity. The aim of this study was to assess the relationship between mood instability and the risk of developing chronic low back pain, using a rigorously designed mendelian randomization methodology.

**Method:**

The study incorporated both univariate and multivariate mendelian randomization to analysis the causal relationship between mood instability and the risk of developing chronic low back pain. The data on mood instability from the Integrative Epidemiology Unit (IEU) opened Genome-Wide Association Studies (GWAS) project (IEU-opened GWAS project). Data on low back pain were collected from two sources: One source is the IEU open GWAS project (discovery data). Another source is a GWAS meta-analysis (replication data). Inverse variance weighted method, weighted median method, MR-Egger regression, and mendelian randomization pleiotropy residual sum and outlier method were used for mendelian randomization analysis.

**Result:**

The univariable mendelian randomization analysis shows a statistically significant correlation between mood instability and the risk of low back pain. Several methods were performed, including inverse variance weighting (discovery data: odds ratio = 3.544, 95% confidence interval = 1.785–7.039, *p* = 0.000; replication data: odds ratio = 3.167, 95% confidence interval = 2.476–4.052, p = 0.000), MR-Egger (discovery data: odds ratio = 7.178, 95% confidence interval = 0.057–909.525, *p* = 0.429; replication data: odds ratio = 2.262, 95% confidence interval = 0.580–8.825, *p* = 0.246), weighted median (discovery data: odds ratio = 2.730, 95% confidence interval = 1.112–6.702, *p* = 0.028; replication data: odds ratio = 3.243, 95% confidence interval = 2.378–4.422, *p* = 0.000), MR-PRESSO (discovery data: odds ratio = 3.544, 95% confidence interval = 1.785–7.039, *p* = 0.001; replication data: odds ratio = 3.167, 95% confidence interval = 2.476–4.052, p = 0.000) methods. The results were consistent across these methods. The results obtained from discovery data are consistent with those obtained from discovery data. In the multivariable mendelian randomization, after adjusting for various covariates such as body mass index, current tobacco smoking, alcohol intake frequency, Total body bone mineral density, and vigorous physical activity, there is a consistent correlation between mood instability and chronic low back pain.

**Conclusion:**

This study provides robust evidence supporting a causal relationship between mood instability and the development of low back pain. Our findings suggest that addressing mood instability may play a crucial role in prevention and management strategies for individuals experiencing low back pain.

## 1. Introduction

Low back pain (LBP) is a major public health concern worldwide, as it is one of the leading causes of motor dysfunction, pain, and even disability in musculoskeletal diseases. With the global aging process accelerating, LBP’s burden on public health will continue to increase (1, 2). LBP patients typically seek medical attention due to pain and functional limitations, but the etiology is often complex and multifactorial, with non-specific risk factors such as aging, muscle and bone decline, unhealthy lifestyle, etc. contributing to recurrent episodes of LBP. These episodes can cause significant physical harm, resulting in frequent medical attention and significant social and economic burdens (3). There are various evidence-based treatment methods available for LBP, including physical therapy, medication, and surgical interventions that can relieve dysfunction and reduce pain. Studies have shown that over 80% of patients can recover within 3 months after treatment (4), but approximately 70% of patients will recur within a year (5). This highlights the importance of prevention strategies in preventing recurrence and exacerbation. Preventive measures can effectively improve patient quality of life and reduce medical burdens (6, 7).

One of the defining features of mood instability (MI) is its rapid and unpredictable changes in emotions and feelings, which can be observed in both healthy individuals and those with mental disorders. MI cannot be used as a standalone diagnostic criterion, so its etiology and treatment plan remain unclear at present. MI is not a single symptom but a multidimensional issue that encompasses various aspects of emotional regulation and expression (8–10). MI can exacerbate other conditions, evidence shows that patients with depression-related LBP experience more severe pain and disability than those with normal emotions, and negative emotional state can lead to noncompliance with treatment and significantly impact therapeutic outcomes (11). On the other hand, LBP-induced pain and disability can also cause significant psychological trauma to patients, resulting in sleep disturbances, anxiety, depression, and fearful behaviors. Limited mobility due to dysfunction can significantly reduce the quality of life of patients. There is a vicious cycle between LBP and MI, greatly affecting patient prognosis and quality of life. A systematic review analyzed the effectiveness of psychological interventions in the treatment of LBP. The study included 2,490 patients and conducted a meta-analysis on the effectiveness of emotion treatment in pain, disability, quality of life, and other MI conditions. The results showed that psychological intervention can effectively reduce pain intensity and improve quality of life. However, due to the lack of appropriate controls, the relationship between MI and LBP has not been systematically analyzed or evaluated, making it uncertain whether MI plays a causal role in the development of LBP (12).

Mendelian randomization (MR) is a technique used to assess the presence of a causal relationship between risk factors and health or disease. It leverages genetic variation as an instrumental variable to mitigate the issue of reverse causation. To ensure validity, MR research requires the identification of genetic variants associated with the exposure under investigation, as well as the testing of associations between these variants and the outcome of interest. To serve as a reliable tool for causal inference in MR studies, a genetic variant must fulfill three fundamental assumptions: Firstly, the instrumental variable SNP should be closely related to the exposure. Secondly, the SNP should not be associated with any confounding factors related to exposure or outcome and should to eliminate the SNPs of linkage disequilibrium. Finally, the SNP should not be a method related to the outcome that is unrelated to the exposure pathway. With MR, the effect of remaining confounding factors on the accuracy of the correlation results is circumvented, making the strength of the correlation result argument reliable.

The use of MR can help establish the causal relationship between exposure and outcomes. By utilizing single nucleotide polymorphisms (SNPs) to predict SNPs associated with MI, we can determine the causal impact of MI on LBP. By using the genetic variant SNP as an instrument, thus effectively avoiding measurement error and reverse causation (genetic factors predate exposure variables in time and are therefore less likely to be associated with confounders), by analyzing genetic variants associated with exposure through MR, and then testing for associations between these genetic variants and outcomes, MR can then be used to estimate the causal relationship between exposure and outcome when the three core assumptions are met (13, 14). Numerous studies have been conducted to explore the relationship between MI and LBP, but many of them are clinical trialls or system reviews with a small sample size (usually <100 participants), and they typically represent single-center studies that may have limited generalizability (15, 16). Moreover, the findings of some of these studies are inconclusive, and there is still no clear consensus. Using large-scale, multicenter, and large-sample GWAS database-based data, MR analysis overcomes the limitations of small sample sizes in previous clinical trials or system reviews, thereby increasing the reliability and generalizability of conclusions. Furthermore, MR analysis is a robust analytical method that avoids confounding, reverse causality, and various forms of bias, thus enabling the inference of a causal relationship between exposure and outcome (17, 18). The application of MR can enhance the identification of potential targets for intervention (e.g., emotional instability), offering an additional rational approach to clinical treatment.

In this study, a two sample MR analysis was employed to uncover the potential causal effect relationship between MI and LBP. Furthermore, the multivariate MR analysis was employed to exclude the influence of confounding factors on LBP. By MR analysis, we avoided causal inversion and also excluded the influence of confounding factors, thus avoiding the bias found in traditional epidemiologic studies in the past and effectively revealing a causal relationship between exposure and outcome rather than simply suggesting an association exposure and outcome.

Our research provides the first evidence of the causal connection between MI and LBP through MR analysis.

## 2. Methods

### 2.1. Ethical approval

Our studies are based on publicly available GWAS data, so no additional ethical approval is required.

### 2.2. Data acquisition

All participants in the GWAS were of European descent and were either male or female. The summary exposure data for MI was obtained from a GWAS study conducted by the UK Biobank (GWAS ID: ukb-b-14,180, N = 451,619).^1^ Participants were genotyped using an Affymetrix UK Biobank Axiom array, and extensive quality control was performed on the genetic data. The outcome data for LBP came from the FinnGen study (GWAS ID: finn-b-M13_LOWBACKPAIN),^2^ and cases of LBP were identified according to International Classification of Diseases (ICD) coding. To perform replication analysis, we also used the largest genetic study of back pain phenotypes, which is a meta-GWAS dataset include four data bank: the deCODE Genetics (Iceland),the Danish Blood Donor Study (Denmark); DBDS and Copenhagen Hospital Biobank; CHB(Denmark), and the UK Biobank; UKB(United Kingdom) (containing 119,100 cases and 909,847 controls), where the “dorsalgia” code group (M54) was used to identify cases of healthcare-associated back pain, which is primarily considered chronic/recurrent back pain (19). The corrected analysis data for multivariable MR summary (body mass index (BMI), current tobacco smoking, alcohol intake frequency, total body bone mineral density, and vigorous physical activity) were obtained from the IEUOpenGWAS project. The effect alleles of MI and LBP datasets were harmonized to confirm that exposure and the effect outcome correspond to the same allele by using the harmonize data function from the TwoSampleMR package.

### 2.3. Instrumental variable selection

To ensure that MR analysis meets the three core assumptions, we perform quality control and quality control techniques on all SNPs. Firstly, we set the correlation hypothesis thresholds (*p* < 5 × 10^−8^, *F* > 10) and exclusion hypothesis thresholds (*p* > 5 × 10^−5^). Secondly, to eliminate the SNPs of linkage disequilibrium, we set the independence hypothesis thresholds (clump r^2^ = 0.001, clump kb = 10,000). To meet the independence hypothesis, we use the PhenoScanner website to retrieve each SNP and ensure exclusion of confounding factors. We also remove SNPs with palindromic structures using the harmonize data function. For the remaining SNPs, the MR-PRESSO test does not detect potential outliers (20, 21). Finally, we select these SNPs that satisfies the threshold setting as instrumental variables for MI assessment. These instrumental SNPs explain 0.39% of MI variations. In our study, the F statistic value of single SNPs ranges from 29.8 to 63.7, with a power of 100%.

### 2.4. Mendelian randomization analysis

The inverse variance weighting (IVW) method was utilized as the primary MR analysis to explore the potential causal relationship between MI and LBP. This study employed the IVW method as the main MR approach, which can obtain the most accurate and reliable causal relationship. Additionally, we also utilized MR-Egger regression, Weighted-Median, Mendelian randomization pleiotropy residual sum and outlier (MR-PRESSO) tools for evaluation and comprehensive assessment of consistency evidence (22). Additionally, the heterogeneity analysis, the pleiotropy analysis, the leave-one-out analysis was conducted to evaluate the robustness of the conclusions, whether there is bias in the outcome, and whether there is a SNP that seriously affects the outcome (20, 23–25). These methods have their advantages and disadvantages, such as that the IVW method is based on all core assumptions of MR being valid assumptions, may have potential horizontal polymorphism effects, and the causal estimates of IVW may be biased. The MR-Egger method can provide unbiased assessments even when violating exclusion restriction assumptions, but its statistical capacity is relatively low. Through the integration of multiple MR methods, the bias of confounding and reverse causality is maximized to improve the accuracy of estimating causal relationships.

### 2.5. Statistical analysis

To conduct the MR analysis, we used the R Studio software (R version 4.2.0) package TwoSampleMR^3^ and MendelianRandomization^4^ (26). The main analysis of MI and LBP utilized the random effect model using the IVW method, the MR-Egger method, Weighted-Median method, MR-PRESSO method for evaluation and comprehensive assessment of consistency evidence. The Cochran’s Q statistical test was used to assay for the presence of pleiotropy. To remove potential confounding factors, the variance backward weighted analysis was conducted. A *p* value of 0.05 was set to indicate statistical significance for tests of pleiotropy and heterogeneity.

## 3. Results

### 3.1. Selection of data sources

The data on MI from the IEU-opened GWAS project (GWAS ID: ukb-b-14,180, a total of 451,619 individuals, including 204,412 cases and 247,207 controls). Data on low back pain were collected from two sources: One source is the IEU open GWAS project (ID: finn-b-M13_LOWBACKPAIN), which included 13,178 cases and 164,682 controls (discovery data), 45 independent genetic variants represent MI. Another source is a GWAS meta-analysis, this dataset includes four data banks: the deCODE Genetics (Iceland), the Danish Blood Donor Study (Denmark); DBDS and Copenhagen Hospital Biobank; CHB(Denmark), and the UK Biobank; UKB (United Kingdom), the “dorsalgia” code group (M54) was used to identify cases of healthcare-associated back pain, which is primarily considered chronic/recurrent back pain. The dataset included 119,100 cases and 909,847 controls (replication data) (Table 1). IVW method, weighted median method, MR-Egger regression, and MR-PRESSO method were used for MR analysis.

**Table 1:** Detailed information on the GWAS datasets used in this MR study.

Dataset type	Item	GWAS ID	Author	Consortium	Year	Population	Sample size	Sex
Exposure	Mood instability	ukb-b-14,180	Ben Elsworth	MRC-IEU	2018	European	451,619. 204,412 cases and 247,207 controls	Males and females
	Body mass index	ukb-b-19,953	Ben Elsworth	MRC-IEU	2018	European	461,460	Males and females
	Current tobacco smoking	ukb-b-223	Ben Elsworth	MRC-IEU	2018	European	462,434	Males and females
	Alcohol intake frequency	ukb-b-5779	Ben Elsworth	MRC-IEU	2018	European	462,346	Males and females
	Total body bone mineral density	ebi-a-GCST005348	Medina-Gomez C	NA	2018	European	56,284	Males and females
	Heavy physical activity	ukb-b-13,184	Ben Elsworth	MRC-IEU	2018	European	460,376. 197,006 cases and 263,370 controls	Males and females
Outcome	Low back pain	finn-b-M13_LOWBACKPAIN	NA	FinnGen	2021	European	13,178 cases and 164,682 controls	Males and females
	Low back pain	NA	Bjornsdottir G	NA	2022	European	119,100 cases and 909,847 controls	Males and females

**Figure 1:**
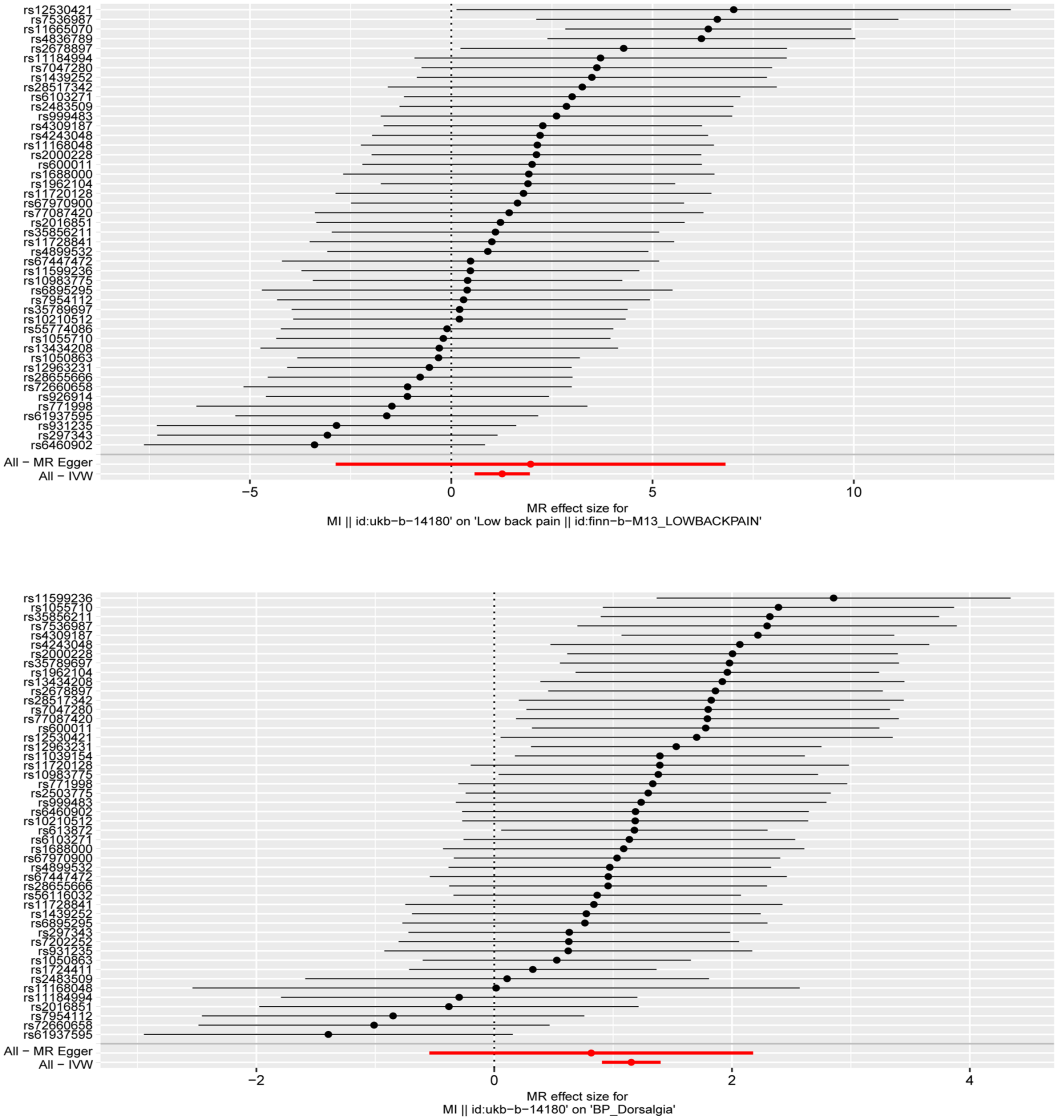
The forest plot was used to show the MR estimate and 95% CI values for each SNP, also show the IVW and MR-Egger MR results at the bottom.

The thresholds ((*p* < 5 × 10^−8^, *F* > 10) was set to satisfy correlation assumptions (Supplementary Table S1). The SNPs related to the ending were excluded (*p* > 5 × 10 ^−5^) to satisfy the exclusionary assumption. The convergence was removed by PhenoScanner website scanning and the chain imbalances was removed by setting thresholds (clump = TRUE, *r* ^2^ < 0.001, kb = 10,000) to satisfy independence assumptions. We also remove SNPs with palindromic structures using the harmonise_data function (action = 2). After satisfying the above three core assumptions of the MR analysis, 46 SNPs was obtained from the discovery data and 48 SNPs was obtained from the replication data. These SNP all have strong potential to predict MI (Figure 1).

### 3.2. MR analysis in LBP

**Figure 2:**
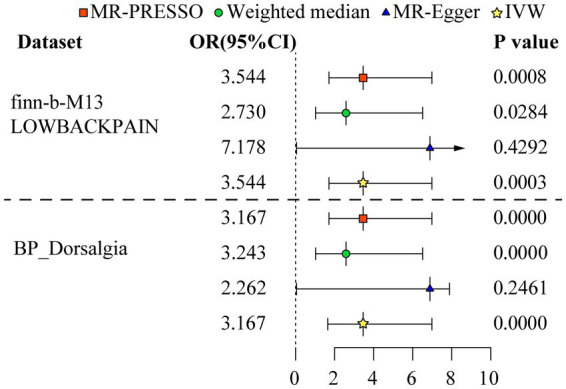
Odds ratio plot for mood instability and low back pain. The four methods applied in the current manuscript were all depicted. Four different colors and shapes of logos represent represent IVW, MR-Egger, weighted median, and MR-PRESSO methods.

The MR analysis was executed to assess whether there was a correlation between MI and LBP. The IVW (discovery data: odds ratio (OR) = 3.544, 95% confidence interval (CI) = 1.785–7.039, *p* = 0.000; replication data: OR = 3.167, 95% CI = 2.476–4.052, p = 0.000), MR-Egger (discovery data: OR = 7.178, 95% CI = 0.057–909.525, *p* = 0.429; replication data: OR = 2.262, 95% CI = 0.580–8.825, *p* = 0.246), weighted median (discovery data: OR = 2.730, 95% CI = 1.112–6.702, *p* = 0.028; replication data: OR = 3.243, 95% CI = 2.378–4.422, p = 0.000), MR-PRESSO (discovery data: OR = 3.544, 95% CI = 1.785–7.039, *p* = 0.001; replication data: OR = 3.167, 95% CI = 2.476–4.052, *p* = 0.000) was executed by TwoSampleMR R package. All these methods demonstrated a robust correlation between MI and LBP. The results of further repeated validation of discovery data and replication data are highly consistent (Figure 2 and Supplementary Table S2).

### 3.3. The sensitivity analysis

**Figure 3:**
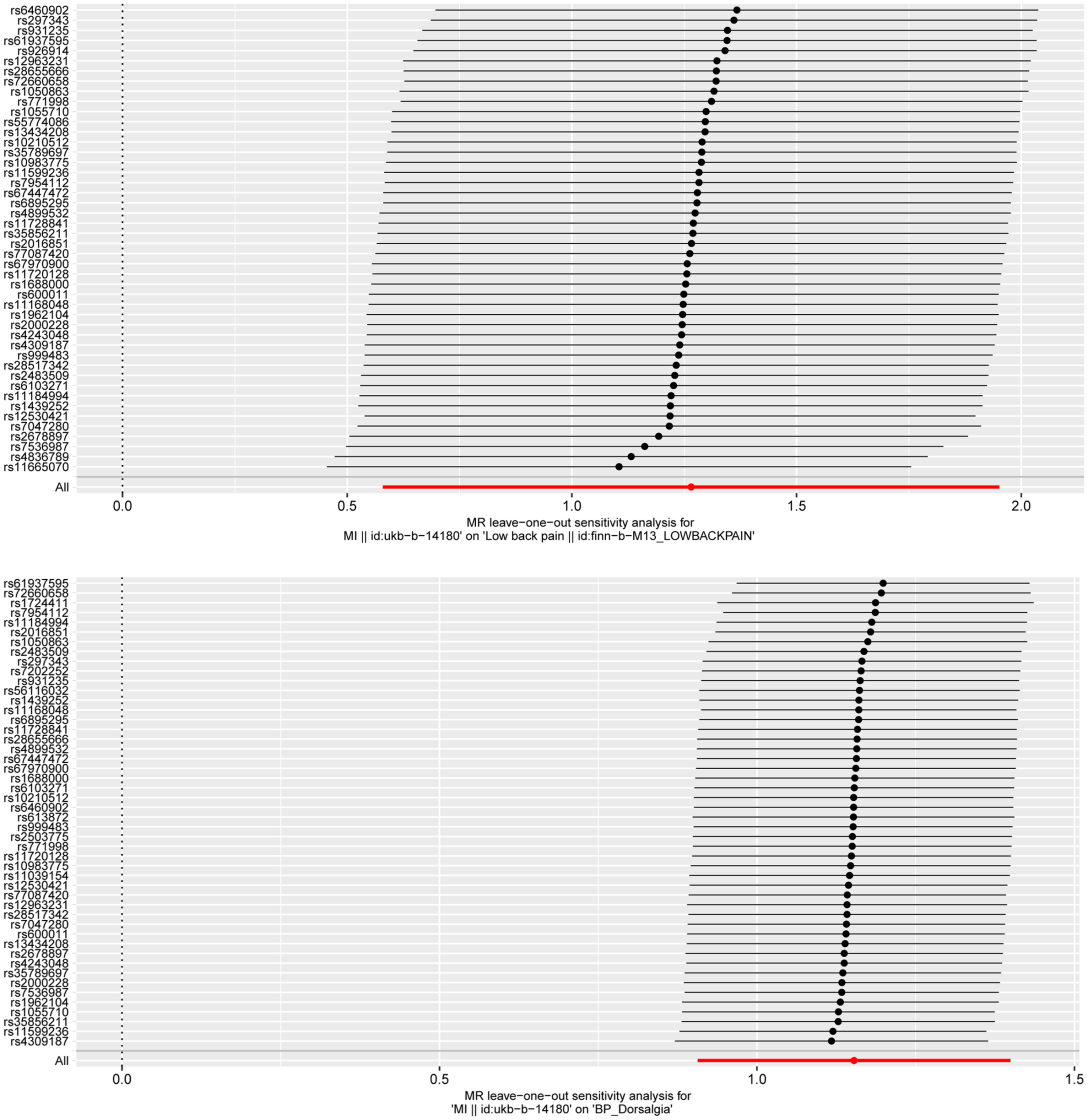
Leave-one-out analyses to evaluate whether any single instrumental variable was driving the causal effect.

**Figure 4:**
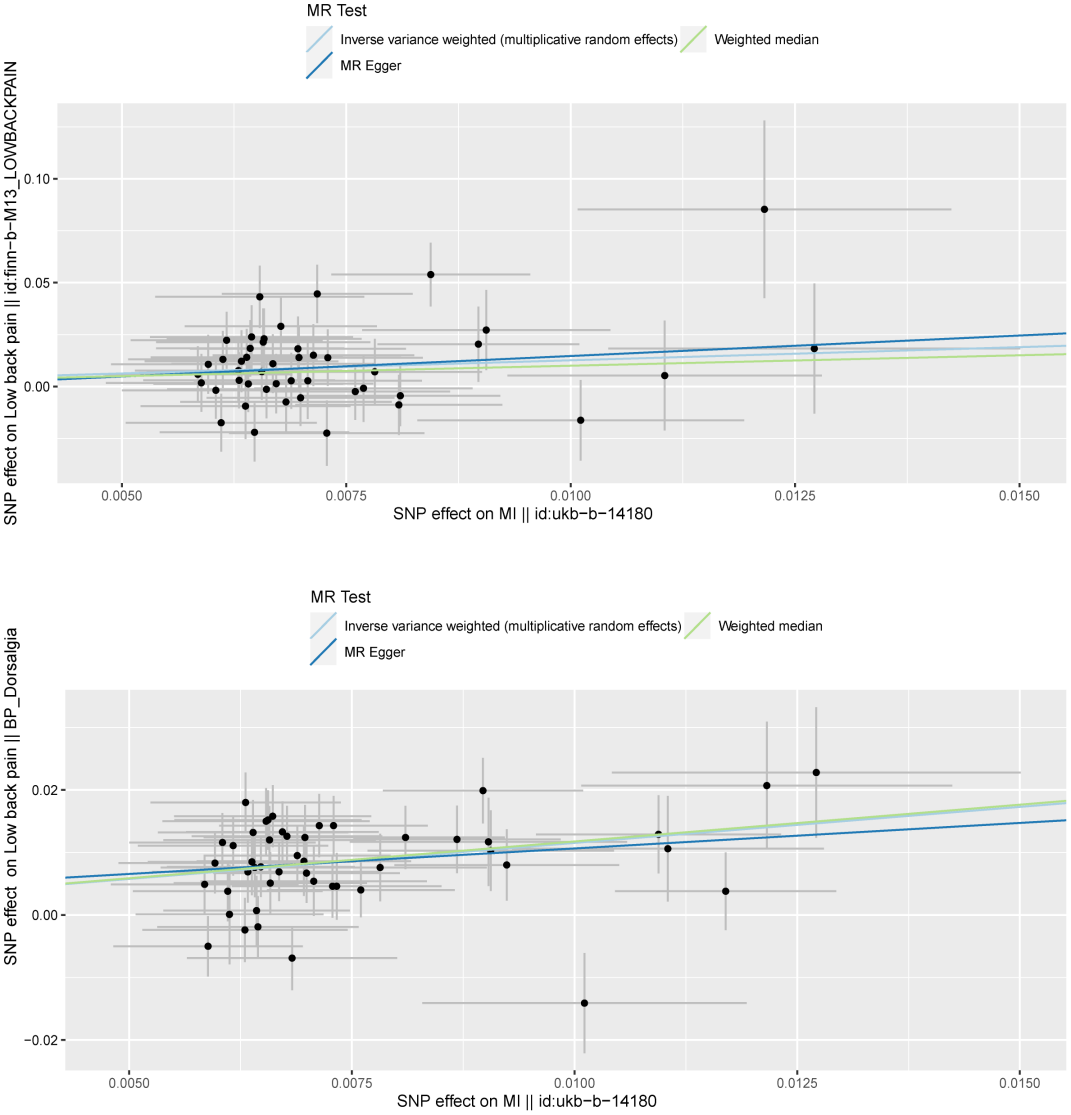
Scatter plot showing the genetic correlations of mood instability and low back pain. The slopes of line represent the causal effect of each method (IVW, Weighted median and MR Egger) respectively.

**Figure 5:**
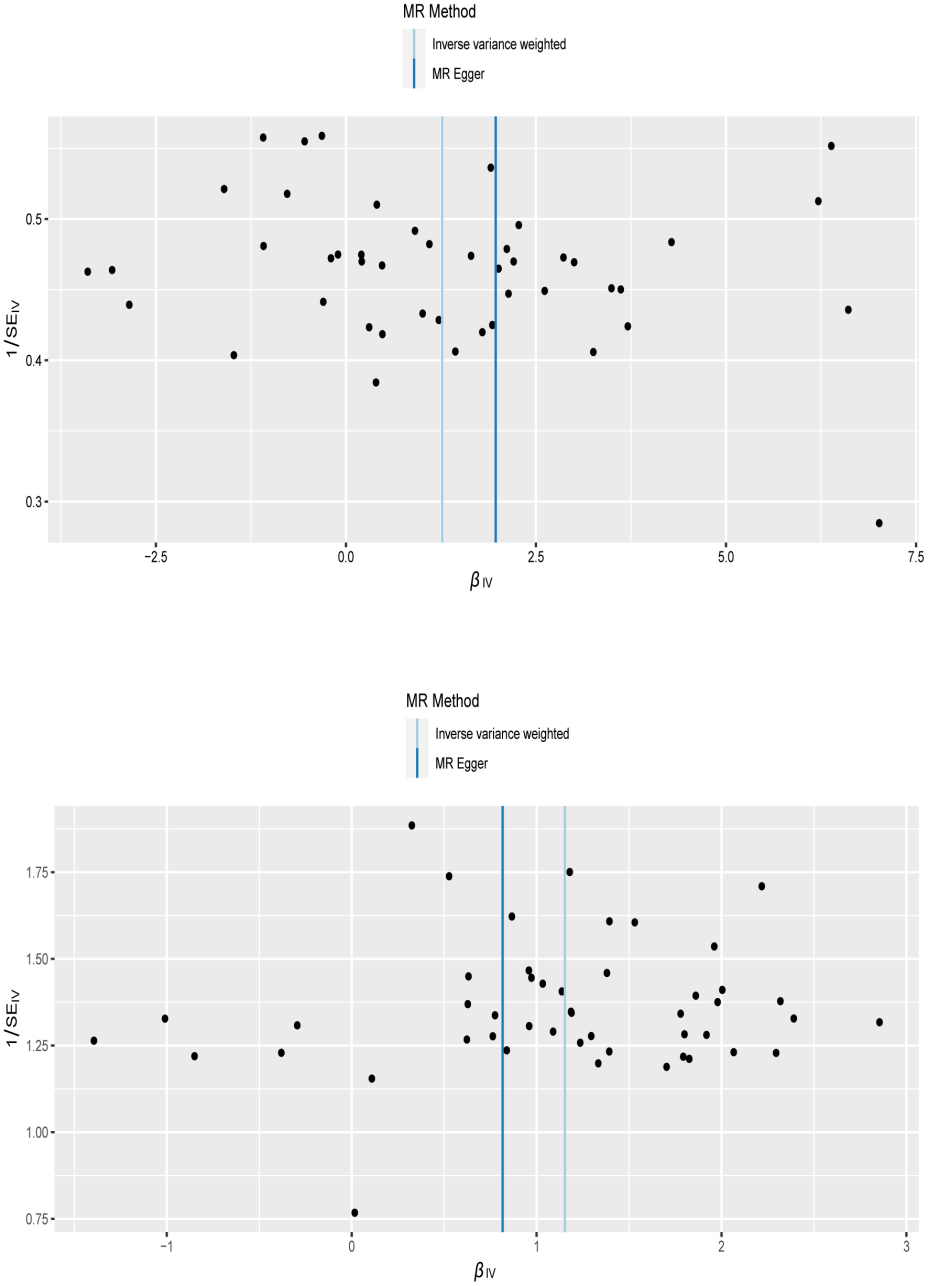
A funnel plot was applied to detect whether the observed association was along with obvious heterogeneity.

To confirm the reliability of the results of the MR analysis, The Cochran’s Q statistical data was calculated to quantify the heterogeneity of individual causal effects, a value of *p* >0.05 indicating the absence of heterogeneity. Both MR-Egger (discovery data: Cochran’s Q = 55.138, *p* = 0.121; replication data: Q = 66.816, *p* = 0.024) and IVW methods (discovery data: Cochran’s Q = 55.242, *p* = 0.141; replication data: Q = 67.169, *p* = 0.028) was carried out and the same result is output. The replication data shows the heterogeneity (*p* < 0.05), but the beta values are in the same direction, therefore they are also considered positive results (27). Similarly, the pleiotropy statistical data was calculated by MR-Egger intercept analysis (discovery data: *p* = 0.774; replication data: *p* = 0.624) and MR-PRESSO analysis (discovery data: *p* = 0.162; replication data: *p* = 0.055), there both value of *p* >0.05 demonstrated the absence of pleiotropy between MI and LBP. The leave-one-out analysis was carried out to calculate the meta effect of remaining SNP and observe whether the results have changed after removing each SNP (Figure 3). After excluding each SNP, all error lines are on the right side of 0 or all error lines are on the left side of 0. The overall error line does not change significantly. The scatter plots of the SNP-outcome associations against the SNP-exposure associations (Figure 4). The funnel plot was drawn for observing whether SNPs are symmetrically distributed on both sides of the IVW line (Figure 5). All four sensitivity analyses confirmed the reliability of the MR analysis.

### 3.4. Multivariate MR

To confirm whether the correlation of MI on LBP is independent of BMI, current tobacco smoking, alcohol intake frequency, total body bone mineral density, and vigorous physical activity, the multivariate MR analysis was conducted. The results of the multivariate MR analysis demonstrate that there is still a correlation between MI and LBP after correcting for other factors (Table 2).

**Table 2:** Results of multivariable MR.

Exposure	Outcome	Adjusted by factor	SNP	beta	se	*p*-value
Mood instability	Low back pain	Body mass index	400	1.318	0.338	9.72E-05
Mood instability	Low back pain	Current tobacco smoking	77	1.417	0.382	2.09E-04
Mood instability	Low back pain	Alcohol intake frequency	121	1.424	0.353	5.55E-05
Mood instability	Low back pain	Total body bone mineral density	121	1.471	0.348	2.39E-05
Mood instability	Low back pain	Heavy physical activity	68	1.647	0.368	7.57E-06

## 4. Discussion

This is the first study to investigate the relationship between MI and LBP using the multiple validation MR method. Our two sample MR analysis revealed a correlation between MI and LBP, MI increases the likelihood of LBP, and the degree of MI is positively correlated with LBP which was consistent after adjusting for BMI, current tobacco smoking, alcohol intake frequency, total body bone mineral density, and vigorous physical activity.

Until now, the evidence on the relationship between MI and LBP is underdeveloped. Previous studies have suggested a link between emotions and LBP (28, 29). LBP is a chronic recurrent pain condition that often causes fear and depression in patients, MI can exacerbate the pain experience, leading to a vicious cycle. Activities that promote emotional stability, such as yoga, may alleviate LBP by improving mood (30). A study utilized breathwork, meditation, and yoga philosophy lectures in LBP patients, resulting in a reduction of anxiety levels by 20.4%, depression levels by 47%, LBP reduction by 49%, improved spinal mobility by 50%, and increased overall well-being (31). Yoga-based physical therapy that enhances mood is more effective in relieving pain than conventional physical therapy (32, 33). Moreover, with continued practice of yoga, the relief of pain and emotional stability persists over time (34). However, some studies have yielded differing results. A systematic review indicated that MI was associated with adverse treatment outcomes in short-term (less than 6 months) low back pain patients but not in long-term LBP (35).

Several potential explanations may account for the heterogeneity observed. Firstly, BMI may serve as a common risk factor for both MI and LBP (36, 37). An increase in BMI can lead to MI and heightened emotional impulses. A MR analysis conducted on patients revealed that an increase of 5 kg/m^2^ in BMI can cause MI, such as depression and anxiety (38). Additionally, an increased load on the lower back is associated with an elevated BMI, as demonstrated by another MR analysis on patients, which revealed that increases in waist circumference, hip circumference, and overall body fat levels can cause degenerative changes in the intervertebral discs and increase the risk of low back pain after adjusting for BMI (39). The correlation between BMI and LBP is significantly weakened when other factors are considered. Secondly, smoking and drinking as risk factors may have contributed to confusion in studying the relationship between MI and LBP (40–42). Furthermore, some genetic variations may be associated with an increased susceptibility to both MI and LBP. Therefore, MI may not be a reliable predictor factor for the development and outcome of LBP. Previous studies have been unable to avoid the influence of these confounding factors. In this study, we effectively eliminated these biases and confounding factors through multi-variate MR analysis and improved research design, thereby providing more credible evidence in support of our findings.

There are several potential pathways that have been widely accepted between MI and LBP at both the biological and behavioral levels. MI can impact endocrine and immune metabolic processes, which can significantly influence the occurrence and development of LBP (43, 44). The endocrine and immune systems are complex networks that are regulated by numerous factors. When emotions are unstable, both the endocrine and immune systems are subject to feedback regulation (45). During periods of emotional instability, hypothalamus neurons release adrenocorticotropin-releasing hormone and arginine vasopressin, which stimulate the release of various hormones from the adrenal glands, leading to changes in the human endocrine system (46, 47). Changes in hormone levels in the endocrine system can also affect the levels of immune cells and inflammatory cytokines, resulting in changes in the immunological microenvironmental homeostasis (48, 49). Additionally, MI can activate central and peripheral immune cells, leading to the release of proinflammatory cytokines, proinflammatory cytokines pass through the blood–brain barrier to reach the brain, creating a vicious cycle, that is consistent with the vicious cycle caused by MI and LBP (50, 51).

In our study, we employed a two sample MR design to assess the causal relationship between MI and LBP. Similar to previous MR studies, we utilized IVW methods in the primary MR analysis. The results of the IVW were statistically significant, indicating that there is no single tool for driving causality estimation using SNPs. Multiple sensitivity analyses were conducted utilizing various methods, and the findings remained consistent and stable. To validate the causal relationships found in the data set, we employed validation samples of LBP from another meta-GWAS dataset. Repeated analyses yielded similar results, establishing a causal link between MI and LBP. Furthermore, to account for potential confounding factors such as BMI, current tobacco smoking, alcohol intake frequency, total body bone mineral density, and vigorous physical activity, we employed multivariate MR. Overall, these pieces of evidence have bolstered the robustness of our research findings.

Our study has several advantages. Firstly, we conducted bidirectional MR analysis and repeated validation to summarize and calculate the causal relationship between MS and LBP, effectively avoiding confounding bias and reverse causality, which are not available in many observational and prospective studies. By eliminating confounding bias and reverse causality, our study provides more robust evidence for the correlation between MI and LBP. Secondly, our research results offer insights into the prevention and treatment of both MI and LBP. Given the high incidence of both conditions in the general population, uncovering the causal relationship between them will help us make more efforts in early prevention and timely intervention. Additionally, recognizing this causal relationship suggests that providing more mental health treatment during the LBP process can bring more clinical benefits to patients. However, our study also has some limitations. Firstly, all GWAS data come from European populations, and it remains to be studied whether the results described are consistent in other populations. Secondly, Because the MI dataset and the LBP dataset we use both have UK Biobank databases (replication data is a meta-GWAS dataset include four data banks, the UK Biobank was among them). So, there is a small overlap between the samples (<10%), which is usually considered acceptable and does not affect the results significantly (21, 52). Thirdly, the replication data shows the heterogeneity (*p* < 0.05), but the beta values are in the same direction, therefore they are also considered positive results. But these results are all available and it can support our conclusions.

## 5. Conclusion

This is the first MR analysis to examine the causal relationship between MI and LBP. Our analysis revealed that MI is associated with an increased risk of developing LBP, which remained consistent after adjusting for BMI, current tobacco smoking, alcohol intake frequency, total body bone mineral density, and vigorous physical activity. This study provides further support for the correlation between MI and LBP and offers valuable insights into the prevention and treatment of LBP.

## Data availability statement

The original contributions presented in the study are included in the article/Supplementary material, further inquiries can be directed to the corresponding authors.

## Ethics statement

Ethical review and approval was not required for the study on human participants in accordance with the local legislation and institutional requirements. Written informed consent from the patients/participants or patients/participants' legal guardian/next of kin was not required to participate in this study in accordance with the national legislation and the institutional requirements.

## Author contributions

RL and RM conceived the idea for the study and provided critical revision of the manuscript. QL participated in study design, data acquisition, and data analysis. SX and RM participated in writing and drafting of the manuscript. All authors contributed to the article and approved the submitted version.

## Funding

The research was supported by the Hospital level scientific research project of Xiangyang Central Hospital (Grant No. 2021C10).

## Conflict of interest

The authors declare that the research was conducted in the absence of any commercial or financial relationships that could be construed as a potential conflict of interest.

## Publisher’s note

All claims expressed in this article are solely those of the authors and do not necessarily represent those of their affiliated organizations, or those of the publisher, the editors and the reviewers. Any product that may be evaluated in this article, or claim that may be made by its manufacturer, is not guaranteed or endorsed by the publisher.

## Footnotes

^1^ https://gwas.mrcieu.ac.uk/datasets/ukb-b-14180/

^2^ https://gwas.mrcieu.ac.uk/datasets/finn-b-M13_LOWBACKPAIN/

^3^ https://github.com/MRCIEU/TwoSampleMR

^4^ https://github.com/sb452/MendelianRandomization/tree/v0.5.0
