# Supplementary material for: Mood instability and low back pain: a mendelian randomization study
Source: Front Neurol. 2023 Sep 15;14:1252329. doi: 10.3389/fneur.2023.1252329 (PMC10541504; doi:10.3389/fneur.2023.1252329)
Supplement: Supplementary file 1 [file Table_1.docx]

**Supplement Table 1. SNPs with strong predictive potential**

| Dataset | finn-b-M13_LOWBACKPAIN | | Dataset | BP_Dorsalgia | |
| --- | --- | --- | --- | --- | --- |
| SNP | R^2^ | F | SNP | R^2^ | F |
| rs10210512 | 8.1481E-05 | 36.8012878 | rs10210512 | 8.15E-05 | 36.80129 |
| rs1050863 | 0.00011492 | 51.9042458 | rs1050863 | 0.000115 | 51.90425 |
| rs1055710 | 7.8987E-05 | 35.674775 | rs1055710 | 7.9E-05 | 35.67478 |
| rs10983775 | 9.5509E-05 | 43.1377604 | rs10983775 | 9.55E-05 | 43.13776 |
| rs11168048 | 7.4721E-05 | 33.747904 | rs11039154 | 0.000123 | 55.62671 |
| rs11184994 | 7.2019E-05 | 32.5274337 | rs11168048 | 7.47E-05 | 33.7479 |
| rs11599236 | 7.7126E-05 | 34.8340352 | rs11184994 | 7.2E-05 | 32.52743 |
| rs11665070 | 0.00012842 | 58.0048307 | rs11599236 | 7.71E-05 | 34.83404 |
| rs11720128 | 6.7281E-05 | 30.3871611 | rs11720128 | 6.73E-05 | 30.38716 |
| rs11728841 | 6.8206E-05 | 30.8049612 | rs11728841 | 6.82E-05 | 30.80496 |
| rs12530421 | 7.5558E-05 | 34.125744 | rs12530421 | 7.56E-05 | 34.12574 |
| rs12963231 | 0.00011712 | 52.8995095 | rs12963231 | 0.000117 | 52.89951 |
| rs13434208 | 7.392E-05 | 33.3861876 | rs13434208 | 7.39E-05 | 33.38619 |
| rs1439252 | 8.1534E-05 | 36.8249657 | rs1439252 | 8.15E-05 | 36.82497 |
| rs1688000 | 7.626E-05 | 34.4429396 | rs1688000 | 7.63E-05 | 34.44294 |
| rs1962104 | 0.00010515 | 47.4927472 | rs1724411 | 0.000197 | 88.8634 |
| rs2000228 | 8.9399E-05 | 40.3777598 | rs1962104 | 0.000105 | 47.49275 |
| rs2016851 | 6.6225E-05 | 29.9101004 | rs2000228 | 8.94E-05 | 40.37776 |
| rs2483509 | 8.3414E-05 | 37.6740992 | rs2016851 | 6.62E-05 | 29.9101 |
| rs2678897 | 8.8645E-05 | 40.0372625 | rs2483509 | 8.34E-05 | 37.6741 |
| rs28517342 | 6.7339E-05 | 30.4134898 | rs2503775 | 7.46E-05 | 33.71099 |
| rs28655666 | 9.8857E-05 | 44.6500557 | rs2678897 | 8.86E-05 | 40.03726 |
| rs297343 | 9.9254E-05 | 44.8293221 | rs28517342 | 6.73E-05 | 30.41349 |
| rs35789697 | 8.5639E-05 | 38.6791591 | rs28655666 | 9.89E-05 | 44.65006 |
| rs35856211 | 8.6181E-05 | 38.9239981 | rs297343 | 9.93E-05 | 44.82932 |
| rs4309187 | 0.00014112 | 63.7425528 | rs35789697 | 8.56E-05 | 38.67916 |
| rs4836789 | 0.00010087 | 45.5601081 | rs35856211 | 8.62E-05 | 38.924 |
| rs4899532 | 9.4514E-05 | 42.6881799 | rs4243048 | 7.91E-05 | 35.72951 |
| rs55774086 | 8.8624E-05 | 40.0276988 | rs4309187 | 0.000141 | 63.74255 |
| rs600011 | 8.3381E-05 | 37.6595249 | rs4899532 | 9.45E-05 | 42.68818 |
| rs6103271 | 9.5049E-05 | 42.9300038 | rs56116032 | 0.000119 | 53.62999 |
| rs61937595 | 6.8352E-05 | 30.8711843 | rs600011 | 8.34E-05 | 37.65952 |
| rs6460902 | 8.3552E-05 | 37.7368403 | rs6103271 | 9.5E-05 | 42.93 |
| rs67447472 | 8.811E-05 | 39.7956503 | rs613872 | 0.00014 | 63.30751 |
| rs67970900 | 7.762E-05 | 35.0570302 | rs61937595 | 6.84E-05 | 30.87118 |
| rs6895295 | 6.862E-05 | 30.9919238 | rs6460902 | 8.36E-05 | 37.73684 |
| rs7047280 | 7.3791E-05 | 33.3275046 | rs67447472 | 8.81E-05 | 39.79565 |
| rs72660658 | 7.4314E-05 | 33.5640921 | rs67970900 | 7.76E-05 | 35.05703 |
| rs7536987 | 6.9849E-05 | 31.5471345 | rs6895295 | 6.86E-05 | 30.99192 |
| rs77087420 | 6.8024E-05 | 30.7231016 | rs7047280 | 7.38E-05 | 33.3275 |
| rs771998 | 6.5935E-05 | 29.7794784 | rs7202252 | 8.57E-05 | 38.70537 |
| rs7954112 | 6.7854E-05 | 30.6459088 | rs72660658 | 7.43E-05 | 33.56409 |
| rs926914 | 0.00010886 | 49.169049 | rs7536987 | 6.98E-05 | 31.54713 |
| rs931235 | 7.299E-05 | 32.9659674 | rs77087420 | 6.8E-05 | 30.7231 |
| rs999483 | 7.4014E-05 | 33.4285348 | rs771998 | 6.59E-05 | 29.77948 |
| rs4243048 | 7.9108E-05 | 35.7295141 | rs7954112 | 6.79E-05 | 30.64591 |
|  |  |  | rs931235 | 7.3E-05 | 32.96597 |
|  |  |  | rs999483 | 7.4E-05 | 33.42853 |
