# Supplementary material for: Mood instability and low back pain: a mendelian randomization study
Source: Front Neurol. 2023 Sep 15;14:1252329. doi: 10.3389/fneur.2023.1252329 (PMC10541504; doi:10.3389/fneur.2023.1252329)
Supplement: Supplementary file 2 [file Table_2.docx]

**Supplement Table 2. MR Analysis in CLBP**

| Dataset | Method | p-val | or | or_lci95 | or_uci95 |
| --- | --- | --- | --- | --- | --- |
| finn-b-M13_LOWBACKPAIN | Inverse variance weighted | 0.0003 | 3.5443 | 1.7846 | 7.0393 |
|  | MR Egger | 0.4292 | 7.1783 | 0.0567 | 909.5246 |
|  | Weighted median | 0.0284 | 2.7302 | 1.1122 | 6.7021 |
|  | MR-PRESSO | 0.0008 | 3.5443 | 1.7846 | 7.0393 |
| BP Dorsalgia | Inverse variance weighted | 0.0000 | 3.1674 | 2.4759 | 4.0521 |
|  | MR Egger | 0.2461 | 2.2617 | 0.5796 | 8.8253 |
|  | Weighted median | 0.0000 | 3.2430 | 2.3783 | 4.4222 |
|  | MR-PRESSO | 0.0000 | 3.1674 | 2.4759 | 4.0521 |
